# Effect of Media Use on HIV/AIDS-Related Knowledge and Condom Use in Sub-Saharan Africa: A Cross-Sectional Study

**DOI:** 10.1371/journal.pone.0068359

**Published:** 2013-07-12

**Authors:** Minsoo Jung, Monisha Arya, Kasisomayajula Viswanath

**Affiliations:** 1 Center for Community-Based Research, Dana-Farber Cancer Institute, Boston, Massachusetts, United States of America; 2 Department of Health Science, Dongduk Women’s University, Seoul, South Korea; 3 Department of Medicine, Section of Infectious Disease, Baylor College of Medicine, Houston, Texas, United States of America; 4 Department of Social and Behavioral Sciences, Harvard School of Public Health, Boston, Massachusetts, United States of America; Alberta Provincial Laboratory for Public Health/University of Alberta, Canada

## Abstract

It is known that the level of HIV/AIDS-related knowledge and the degree of condom use varies by socioeconomic status (SES). However, there is limited research on the effect of mass media use on HIV/AIDS-related cognitive and behavioral outcomes in low-income countries and how it might influence the association between SES and HIV-related outcomes. We investigated the moderating effect of media use on the relationship between SES and HIV/AIDS-related knowledge and condom use in sub-Saharan Africa in terms of communication inequalities. Cross-sectional data from the Demographic Health Surveys from 13 sub-Saharan countries (2004–10) were pooled. Gender-stratified multivariable poisson regression of 151,209 women and 68,890 men were used to calculate adjusted relative ratios and 95% confidence intervals for the associations between SES, media use, HIV-related outcomes, and condom use. We found significant disparities in mass media use among people from different SES groups as well as among countries. Education and wealth are strongly and positively associated with awareness of HIV/AIDS and knowledge about transmission and prevention of HIV/AIDS and are significantly associated with condom use. These associations are attenuated when the use of various types of mass media is added to the models, with newspapers showing the strongest effect. The findings of this study suggest that media use has the potential to blunt the impact of socioeconomic status though not completely eliminate it. Thus, we need to pay attention to reducing communication inequalities among social groups and countries to moderate the effect of wealth and SES on HIV/AIDS.

## Introduction

Two-thirds of all people infected with HIV live in sub-Saharan Africa, although this region contains little more than 12 percent of the world’s population [1]. In particular, 31% of new HIV infections in the same year occurred in the 10 countries in southern Africa [2]. The devastating impact of HIV/AIDS spans life-expectancy, public health and the economy through its effects on labor productivity [3]. Under resource-poor conditions, using the media to increase HIV/AIDS-related knowledge and promoting condom use may be important for stopping the spread of HIV/AIDS [2]. In fact, the level of HIV/AIDS-related knowledge and the degree of condom use vary according to socioeconomic status (SES) [4], [5]. Little is known about their associations in sub-Saharan Africa. Our hypothesis is that the degree of media use mediates the relationship between SES and HIV/AIDS-related knowledge and condom use [6], [7].

**Figure 1:**
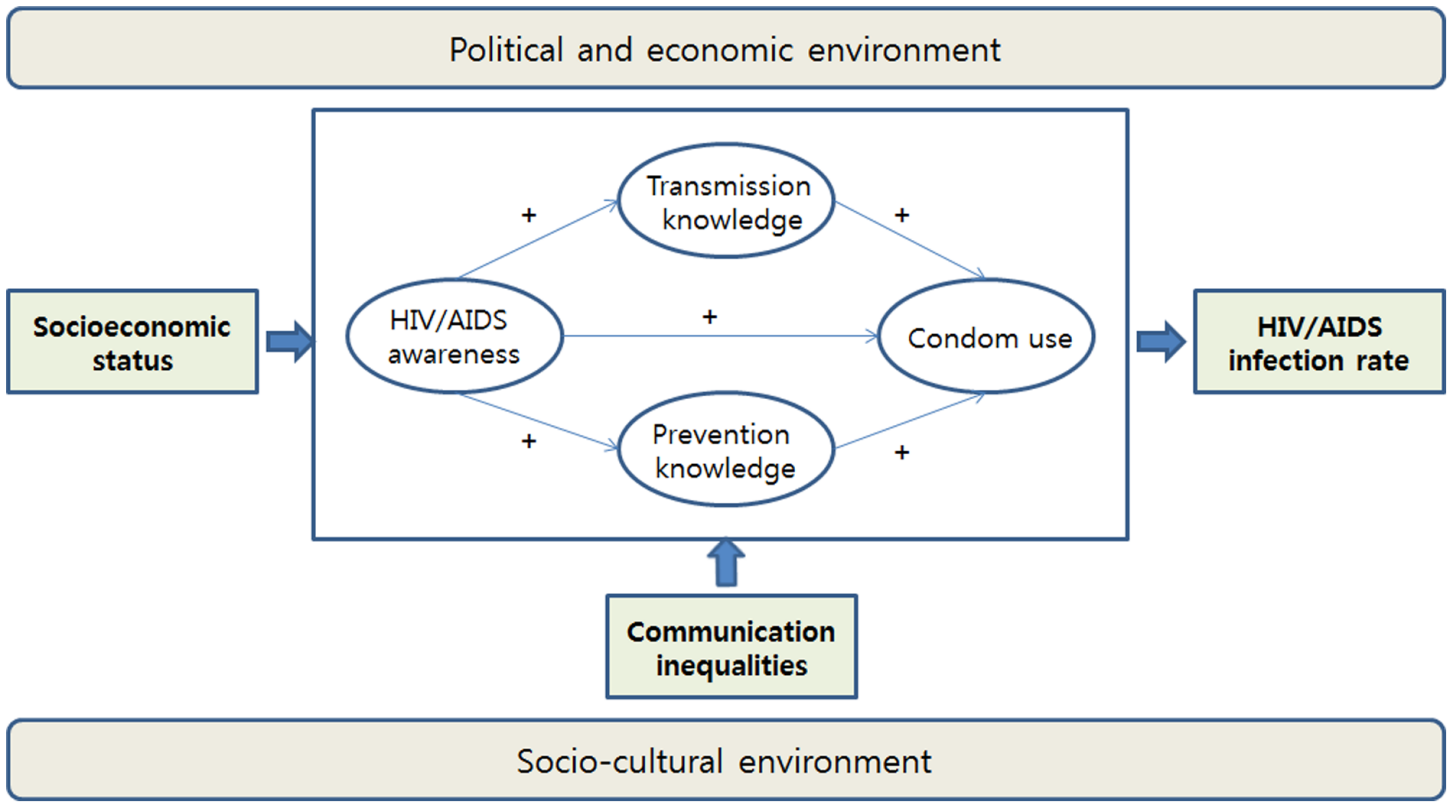
The conceptual frame of this study.

The importance of mass media in health promotion and disease prevention is well documented, since both routine exposure to and strategic use of mass media play a significant role in promoting awareness, increasing knowledge and changing health behaviors [7]–[9]. Accordingly, mass media campaigns have been reliably linked to an increase in HIV/AIDS knowledge among individuals in low-income countries [8], including an awareness of HIV/AIDS, the ways in which the virus is transmitted, and preventive behaviors [10]. Knowledge is an important determinant in the pathways to changing health behaviors [11]. In the case of HIV/AIDS, a high level of awareness is likely to promote safe sex practices such as the regular use of condoms, which may reduce the prevalence rate of HIV infection [4], [12]. Those paths, however, are embedded in an individual’s socioeconomic status as well as in their social and political context (***Figure 1***) [11].

While the contribution of media use to disease prevention and to the promotion of healthy behavior is widely acknowledged [7]–[9], there is mounting evidence of disparities in health communication, characterized as communication inequalities, among different social groups [13]. Although mass media channels such as radio, television and newspapers are important sources of information about HIV/AIDS [11], media-poor groups do not have easy access to these channels [14]. In fact, individuals of lower socioeconomic status (SES) tend to gain less benefit from information flows than their counterparts of higher SES [13]. Therefore, an understanding of inequalities in health communication may contribute to a mass media campaign for population-based approaches that could address the spread of HIV/AIDS worldwide, particularly in low-income countries (LICs). Also, media use may blunt the impact of social inequalities in sub-Saharan African countries. We thus focused on this thread linking SES, the media and HIV/AIDS-related knowledge and behavior.

Sub-Saharan Africa has lagged behind in mass media use and telecommunications during the last 50 years, since gaining independence from Western colonial rule. However, the use of media has spread rapidly since the start of the twenty-first century [15]. Radio is the major source of information in sub-Saharan Africa, and television is the second major source of information [16]. The region saw impressive growth in the number of TV sets owned as well as in the number of television channels available, drawing the attention of international media players as a top emerging territory [17]. In sub-Saharan Africa, 5.2% of all households now have a television set with over 50 major pay-TV service platforms, and 36.9% of the population subscribes to mobile services [18]. The expansion of media provides an important opportunity to address HIV/AIDS-related knowledge and preventive behaviors.

As limited research on health communication inequalities has been conducted in sub-Saharan Africa, the purpose of this study is to use nationally representative surveys of men and women from thirteen African countries to investigate the effect of media use on HIV/AIDS-related knowledge and behavior. Our hypothesis is that communication inequalities, differential media use among social classes, may be one plausible mechanism through which social inequalities in wealth and education lead to knowledge disparities of HIV/AIDS in sub-Saharan Africa.

## Methods

### Ethics Statement

Approval for the study was granted by the Harvard School of Public Health Institutional Review Board (April 27, 2012). All participants gave written informed consent to participate. The Ethics Committees of the Demographic Health Survey approved this consent procedure. During the data collection process, any information that could distinguish individual respondents was not collected.

### Dataset

All of the data used in this study were collected by Demographic and Health Surveys (DHS) conducted in various sub-Saharan African countries from 2004 to 2010. The DHS are nationally representative cross-sectional household surveys that use a standardized questionnaire to facilitate comparisons among countries, particularly for low-income settings [19], [20]. Respondents were recruited using a multistage sampling procedure that stratified all the states of each country into urban and rural areas, with each state’s sample size determined in proportion to the size of the state’s urban and rural populations and in consideration of each state’s gender ratio. Primary sampling units, conceptualized as neighborhoods for the purposes of this research, were defined as census enumeration blocks in urban areas and as villages or village clusters in rural areas. These neighborhoods were selected within each state according to the probability proportional to the population size. From the sampling units, the 220,099 respondents, men and women 15 years of age and older, were proportionally selected by gender and age based on the census data. Face-to-face interviews were conducted on the topics of mass media use, HIV/AIDS knowledge, and condom use. The response rates of all the countries were more than 90%. To generate a large dataset with sufficient statistical power to investigate the effect of media use on HIV/AIDS-related knowledge and condom use, we pooled data from the most recent survey in thirteen countries in sub-Saharan Africa since 2004. All of the surveys were carried out under the direction of the government of each country.

### Measures

The measurement indicators of the DHS were developed by the United States Agency for International Development [19].

#### Dependent variables

Four binary variables were created using a methodology developed by the Joint United Nations Program on HIV/AIDS to measure knowledge and behavior about HIV/AIDS: awareness, prevention knowledge, transmission knowledge, and condom use [21]. The HIV/AIDS awareness variable was created using the question, “Have you ever heard of an illness called AIDS?” and allowed for a binary yes/no response. Individuals were considered to be knowledgeable about HIV/AIDS transmission if they responded to three distinct questions by indicating that (1) mosquito bites and (2) sharing food could not spread HIV/AIDS, and that (3) it was possible for a healthy-looking person to have HIV/AIDS. Individuals were considered to be knowledgeable about HIV/AIDS prevention if they responded affirmatively to each of three questions indicating that an individual could reduce their risk of contracting HIV/AIDS if (1) they abstained from sexual intercourse, (2) had only one uninfected sexual partner, and (3) used a condom every time they had sex. Condom use was assessed with a single-item question, “The last time you had sexual intercourse was a condom used?” with response options of yes or no.

#### Independent variables

Media use: Individuals reported how often they used three mass media sources–radio, television, and newspaper–with possible answers being “almost every day” (4), “at least once a week” (3), “less than once a week” (2), and “not at all” (1).

Socioeconomic status: Wealth was defined using an established methodology developed for the sub-Saharan Africa context in which each individual was assigned a wealth score created by weighting responses to 33 questions regarding household possessions and characteristics such as quality of housing, ownership of land, and possession of an automobile with a factor analysis procedure and dividing the results into quintiles [22]. Educational attainment was assessed on the basis of “have you ever attended school” and “what is the highest level of school you attended” with responses categorized as No education, Incomplete primary (Education in single years: 1–6), Complete primary (7), Incomplete secondary (8–11), Complete secondary (12), and Higher (over 13).

#### Potential confounders

Sociodemographic confounders were selected based on a theoretically [23] and empirically [24] defined relationship with HIV/AIDS knowledge and behavior. The type of place of residence categorized as belonging to large cities (national capitals and places with over 1 million population), small cities (urban areas between 50,000 and 1 million population), towns (urban areas less than 50,000 people), and villages (rural areas). The literacy level was measured by asking the respondent “(show card to respondents) can you read whole or any part of this sentence to me?” which was collapsed into categories of Cannot read at all, Able to read only parts of sentence, and Able to read whole sentence. Occupation was created from self-reported jobs and was categorized as not working, performing manual work, performing agricultural work, or performing non-agricultural manual work. We considered occupation as a confounder, not an independent variable, due to the characteristics of the industrial structure of sub-Saharan Africa countries. Two marital status classifications were single or married couple including cohabitation. Age was divided into the groups of 15–19, 20–24, 25–29, 30–34, 35–39, 40–44, 45–49, 50–54, and 55 or older.

### Statistical Analysis

Each binary HIV/AIDS-related outcome was modeled with a modified Poisson regression by gender using STATA version 10.0 (StataCorp, College Station, TX) for providing an accurate relative risk and 95% confidence interval while simultaneously accounting for clustering within neighborhoods [25]. This is because a Poisson regression is necessary when the dependent variable *Y* has a Poisson distribution, and it assumes the logarithm of its expected value can be modeled by a linear combination of unknown parameters. Survey weights were applied to each model to account for multiple adjustments in the sampling procedure to ensure that sample was representative of each sub-Saharan African country. Features of complex sampling design were taken into account using Stata’s svy suite of commands.

## Results

As detailed in Table 1, a total of, 220,099 sub-Saharan Africans were recruited for the study. The largest sample was from Nigeria (*n = *48,871), and the smallest was from Swaziland (*n* = 9,143). On average, over 90% of the respondents were aware of what HIV/AIDS is (Men: 96%; Women: 93%), but a smaller proportion had knowledge about HIV/AIDS transmission (M: 61%; W: 61%) and prevention (M: 64%; W: 68%). Practice of condom use (M: 22%; W: 10%) was much lower than the level of HIV/AIDS knowledge. Only 1–2 people out of ten had used a condom during their last sexual intercourse. Regarding the practice of condom use, the scores for men were two times higher than those of women, which is in line with the literature [26]. The variation in knowledge levels among countries was highest regarding prevention knowledge.

**Table 1:** Distribution of HIV/AIDS-related knowledge and condom use by sub-Saharan African countries (%).

	Country	n	HIV rates	HIV/AIDS awareness			HIV/AIDS transmission knowledge		HIV/AIDS prevention knowledge		Last intercourse used condom	
				Men	Women		Men	Women	Men	Women	Men	Women
Central	Cameroon (‘04)	15936	5.3	99.2		97.8	53.8	51.3	25.4	17.7	29.7	15.2
	Rwanda (‘10)	20000	2.9	99.9		99.9	70.7	73.1	77.1	82.2	13.4	8.1
East	Tanzania (‘10)	12666	5.6	99.8		99.6	71.3	73.3	76.6	78.4	22.2	12.1
	Uganda (‘06)	11034	6.5	99.9		99.2	59.8	56.8	85.0	76.1	17.2	7.9
Southern	Malawi (‘04)	14959	11.0	99.5		98.6	70.0	61.3	9.4	8.2	15.1	5.2
	Namibia (‘06)	13719	13.1	99.1		98.8	69.7	77.2	79.8	80.1	56.9	40.9
	Swaziland (‘06)	9143	25.9	99.3		99.8	70.2	72.3	83.8	87.3	49.3	37.2
	Zambia (‘07)	13646	13.5	99.5		99.0	62.6	60.7	66.4	68.6	22.0	11.9
	Zimbabwe (‘05)	16082	14.3	99.2		97.9	68.4	69.8	67.8	62.9	24.4	8.3
West	Niger (‘06)	12772	0.8	94.7		83.1	7.7	27.7	77.6	78.6	3.4	0.4
	Nigeria (‘08)	48871	3.6	93.2		88.3	63.7	59.7	75.7	65.6	18.6	7.0
	Senegal (‘10)	20617	0.9	97.1		95.3	50.0	46.4	89.0	83.7	18.9	3.7
	Sierra Leone (‘08)	10654	1.6	82.2		69.6	54.3	49.9	71.2	63.7	8.9	2.5
Pooled total	Average Standard deviation	220099	8.1	96.8 (±4.8)		93.8 (±8.6)	61.5 (±16.4)	61.1 (±13.3)	68.1 (±22.9)	64.2 (±23.7)	22.5 (±14.4)	10.3 (±12.1)

Note: Sample weight % used; unweighted count data (n) presented.

The descriptive characteristics of the sample from Table 2 indicate that the most frequently used media type was radio, to which 36.7% listened daily, whereas, only 17.6% and 6.4% watched television and read newspapers daily. Notably, only about 21.7% of the respondents did not use a radio at all compared to 54.4% for television and 65.0% for newspapers.

**Table 2:** General characteristics of the sample.

	*N*	Weighted %		*N*	Weighted %
**Gender**			**Wealth**		
Men	68890	31.3	1^st^ (lowest) quintile	40666	17.2
Women	151209	68.7	2^nd^ quintile	40966	17.9
			3^rd^ quintile	43911	19.3
**Age (years)**			4^th^ quintile	46037	21.6
15–19	47899	21.4	5^th^ (highest) quintile	48519	23.9
20–24	41498	18.9			
25–29	37267	17.2	**Educational attainment**		
30–34	29297	13.4	No education	60253	26.8
35–39	24071	11.0	Incomplete primary	52558	23.6
40–44	18883	8.5	Complete primary	26926	12.8
45–49	16060	7.2	Incomplete secondary	55515	24.7
50–54	3203	1.5	Complete secondary	14571	7.2
55 or older	1921	0.9	Higher	10276	5.0
**Marital status**			**Radio use**		
Single	88050	40.0	Not at all	48840	21.7
Married couple	132049	60.0	Less than once a week	29435	13.4
			At least once a week	61575	28.2
**Location**			Daily	79802	36.7
Rural	147524	65.3			
Urban	72575	34.7	**Television use**		
			Not at all	122547	54.4
**Occupation**			Less than once a week	26845	11.9
None	75136	33.9	At least once a week	33423	16.1
Manual	64187	30.5	Daily	36876	17.6
Agricultural	70158	31.5			
Non- agricultural and non-manual	8804	4.0	**Newspaper use**		
			Not at all	145181	65.0
**Literacy level**			Less than once a week	32544	14.9
Cannot read at all	77568	34.9	At least once a week	28635	13.7
Able to read partially	18021	8.3	Daily	13044	6.4
Able to read perfectly	122226	56.9			
			**Total**	220099	100.0

Note: Sample weight % used; unweighted count data (n) presented.

Both socioeconomic status and media use indicators were positively associated with each other (See ***Appendix S1 in File S1***), and they were consistently associated with HIV/AIDS knowledge and condom use (See ***Appendix S2 in File S1***). Based on these associations, we modeled the multivariate Poisson regression.

HIV/AIDS Awareness: Women in the wealthiest quintile (RR = 3.56; 95% CI: 3.16–4.02) and having 13 or more years of education (RR = 12.12; 95% CI: 7.52–19.54) were most likely to have heard of HIV/AIDS compared to those in the lowest wealth quintile and those with no education, respectively. These relationships were attenuated (RR = 2.27; 95% CI: 1.99–2.58 and RR = 8.68; 95% CI: 5.34–14.11, respectively) after adjusting for mass media use (***Table 3***). Daily radio listening was the medium most strongly associated with HIV/AIDS awareness (RR = 2.30; 95% CI: 2.14–2.48). Similar results were found for men as well (***Table 4***).

**Table 3:** Relative risk (RR) and 95% confidence interval (CI) of socioeconomic and media use characteristics with HIV/AIDS-related knowledge and condom use among sub-Saharan Africa countries in the 2004∼2010 Demographic Health Survey (Women).

	HIV/AIDS awareness				HIV/AIDS transmission knowledge				HIV/AIDS prevention knowledge				Last intercourse used condom			
	Model 1		Model 2		Model 1		Model 2		Model 1		Model 2		Model 1		Model 2	
	RR	95% CI	RR	95% CI	RR	95% CI	RR	95% CI	RR	95% CI	RR	95% CI	RR	95% CI	RR	95% CI
Wealth																
1^st^ (lowest) quintile	1.00		1.00		1.00		1.00		1.00		1.00		1.00		1.00	
2^nd^ quintile	**1.30**	**1.23–1.38**	**1.17**	**1.10–1.24**	**1.17**	**1.13–1.23**	**1.16`**	**1.11–1.21**	1.04	0.99–1.09	1.01	0.96–1.06	**1.27**	**1.15–1.40**	**1.22**	**1.11–1.35**
3^rd^ quintile	**1.55**	**1.45–1.66**	**1.26**	**1.18–1.35**	**1.20**	**1.15–1.25**	**1.16**	**1.11–1.21**	**1.10**	**1.05–1.15**	1.04	0.99–1.09	**1.42**	**1.30–1.56**	**1.32**	**1.20–1.45**
4^th^ quintile	**2.10**	**1.94–.2.28**	**1.52**	**1.40–1.66**	**1.40**	**1.34–1.46**	**1.31**	**1.25–1.37**	**1.15**	**1.10–1.21**	1.06	1.01–1.18	**1.81**	**1.65–1.99**	**1.58**	**1.43–1.74**
5^th^ (highest) quintile	**3.56**	**3.16–4.02**	**2.27**	**1.99–2.58**	**1.87**	**1.78–1.97**	**1.67**	**1.58–1.76**	**1.17**	**1.10–1.24**	1.06	0.99–1.13	**2.00**	**1.80–2.22**	**1.61**	**1.44–1.80**
Education (yrs)																
No education (0)	1.00		1.00		1.00		1.00		1.00		1.00		1.00		1.00	
Incomplete primary (1–6)	**1.82**	**1.67–1.98**	**1.73**	**1.59–1.89**	**0.89**	**0.85–0.93**	**0.89**	**0.85–0.93**	1.00	0.95–1.05	1.00	0.95–1.05	**1.53**	**1.37–1.71**	**1.52**	**1.36–1.71**
Complete primary (7)	**2.29**	**2.06–2.55**	**2.12**	**1.90–2.36**	**1.09**	**1.03–1.16**	**1.08**	**1.02–1.14**	**1.09**	**1.02–1.16**	1.08	1.01–1.15	**1.73**	**1.52–1.97**	**1.68**	**1.48–1.92**
Incomplete secondary (8–11)	**2.73**	**2.31–3.22**	**2.47**	**2.09–2.93**	**1.42**	**1.33–1.51**	**1.35**	**1.27–1.44**	**1.19**	**1.11–1.27**	**1.17**	**1.09–1.25**	**2.33**	**2.03–2.67**	**2.17**	**1.89–2.49**
Complete secondary (12)	**4.69**	**3.70–5.94**	**3.80**	**2.99–4.85**	**1.70**	**1.57–1.84**	**1.57**	**1.45–1.71**	1.09	1.00–1.19	1.07	0.98–1.16	**2.78**	**2.39–3.24**	**2.47**	**2.12–2.88**
Higher (13+)	**12.12**	**7.52–19.54**	**8.68**	**5.34–14.11**	**2.70**	**2.45–2.98**	**2.35**	**2.13–2.60**	**1.14**	**1.04–1.26**	1.10	1.00–1.21	**3.39**	**2.89–3.97**	**2.85**	**2.42–3.45**
Radio Use																
Not at all			1.00				1.00				1.00				1.00	
Less than once a week			**1.94**	**1.80–2.08**			0.97	0.92–1.01			1.04	1.00–1.10			**1.15**	**1.05–1.26**
At least once a week			**2.51**	**2.34–2.69**			**1.08**	**1.04–1.13**			**1.22**	**1.16–1.27**			**1.17**	**1.07–1.27**
Daily			**2.30**	**2.14–2.48**			**1.07**	**1.02–1.11**			**1.20**	**1.15–1.25**			**1.23**	**1.14–1.32**
Television Use																
Not at all			1.00				1.00				1.00				1.00	
Less than once a week			**1.54**	**1.38–1.72**			1.03	0.98–1.08			1.05	1.00–1.11			**1.12**	**1.03–1.22**
At least once a week			**1.49**	**1.34–1.67**			**1.13**	**1.08–1.19**			**1.13**	**1.08–1.19**			**1.20**	**1.10–1.30**
Daily			**1.20**	**1.05–1.37**			**1.11**	**1.06–1.17**			1.00	0.95–1.05			**1.22**	**1.12–1.32**
Newspaper use																
Not at all			1.00				1.00				1.00				1.00	
Less than once a week			**1.55**	**1.30–1.85**			**1.16**	**1.11–1.21**			1.01	0.97–1.06			**1.20**	**1.12–1.29**
At least once a week			1.25	1.03–1.52			**1.30**	**1.24–1.36**			1.04	0.99–1.10			**1.33**	**1.24–1.43**
Daily			1.03	0.73–1.45			**1.31**	**1.22–1.41**			1.09	1.01–1.17			**1.28**	**1.16–1.42**

Note: All models are additionally adjusted for age, gender, marital status, rural/urban location, literacy level, occupation, countries and survey year.

All the figures in bold have a p-value of <0.01.

**Table 4:** Relative risk (RR) and 95% confidence interval (CI) of socioeconomic and media use characteristics with HIV/AIDS-related knowledge and condom use among sub-Saharan Africa countries in the 2004∼2010 Demographic Health Survey (Men).

	HIV/AIDS awareness				HIV/AIDS transmission knowledge				HIV/AIDS prevention knowledge				Last intercourse used condom			
	Model 1		Model 2		Model 1		Model 2		Model 1		Model 2		Model 1		Model 2	
	RR	95% CI	RR	95% CI	RR	95% CI	RR	95% CI	RR	95% CI	RR	95% CI	RR	95% CI	RR	95% CI
Wealth																
1^st^ (lowest) quintile	1.00		1.00		1.00		1.00		1.00		1.00		1.00		1.00	
2^nd^ quintile	**1.27**	**1.13–1.42**	1.07	0.95–1.21	1.07	1.00–1.14	1.05	0.99–1.12	1.09	1.02–1.16	1.06	0.99–1.13	**1.23**	**1.11–1.37**	**1.16**	**1.04–1.29**
3^rd^ quintile	**1.58**	**1.38–1.80**	**1.20**	**1.05–1.38**	**1.20**	**1.13–1.27**	**1.18**	**1.11–1.25**	**1.11**	**1.04–1.19**	1.07	1.01–1.15	**1.44**	**1.30–1.59**	**1.31**	**1.18–1.46**
4^th^ quintile	**1.86**	**1.58–2.19**	**1.27**	**1.07–1.52**	**1.36**	**1.27–1.45**	**1.31**	**1.22–1.40**	**1.18**	**1.10–1.26**	**1.12**	**1.04–1.21**	**1.67**	**1.50–1.85**	**1.43**	**1.28–1.59**
5^th^ (highest) quintile	**3.46**	**2.66–4.51**	**2.07**	**1.57–2.75**	**1.71**	**1.59–1.85**	**1.61**	**1.49–1.75**	1.10	1.01–1.19	1.04	0.95–1.13	**2.05**	**1.82–2.30**	**1.65**	**1.46–1.87**
Education (yrs)																
No education (0)	1.00		1.00		1.00		1.00		1.00		1.00		1.00		1.00	
Incomplete primary (1–6)	**1.62**	**1.39–1.89**	**1.40**	**1.20–1.63**	**0.84**	**0.78–0.91**	**0.85**	**0.79–0.91**	0.98	0.91–1.07	0.98	0.91–1.06	**1.57**	**1.38–1.78**	**1.54**	**1.35–1.75**
Complete primary (7)	**2.04**	**1.70–2.44**	**1.71**	**1.42–2.06**	1.10	1.01–1.20	1.09	1.00–1.19	1.03	0.94–1.13	1.03	0.94–1.14	**1.84**	**1.58–2.13**	**1.79**	**1.54–2.08**
Incomplete secondary (8–11)	**2.94**	**2.27–3.80**	**2.29**	**1.76–2.99**	**1.53**	**1.39–1.67**	**1.48**	**1.35–1.63**	**1.18**	**1.07–1.30**	**1.17**	**1.06–1.29**	**2.26**	**1.94–2.63**	**2.09**	**1.79–2.43**
Complete secondary (12)	**5.64**	**3.96–8.03**	**3.96**	**2.74–5.73**	**2.17**	**1.94–2.43**	**2.04**	**1.82–2.29**	**1.16**	**1.03–1.30**	1.16	1.03–1.30	**2.81**	**2.37–3.34**	**2.46**	**2.06–2.93**
Higher (13+)	**11.98**	**6.48–22.17**	**7.91**	**4.18–14.95**	**3.16**	**2.80–3.56**	**2.90**	**2.56–3.28**	**1.27**	**1.13–1.44**	**1.28**	**1.13–1.45**	**3.38**	**2.85–4.01**	**2.89**	**2.42–3.45**
Radio Use																
Not at all			1.00				1.00				1.00				1.00	
Less than once a week			**2.20**	**1.89–2.55**			0.91	0.84–0.98			**1.26**	**1.16–1.37**			0.98	0.86–1.12
At least once a week			**3.03**	**2.66–3.45**			1.02	0.95–1.09			**1.23**	**1.15–1.33**			1.11	0.99–1.25
Daily			**3.12**	**2.75–3.54**			1.08	1.01–1.15			**1.32**	**1.23–1.41**			**1.35**	**1.21–1.50**
Television Use																
Not at all			1.00				1.00				1.00				1.00	
Less than once a week			**1.47**	**1.23–1.74**			0.97	0.92–1.03			1.00	0.94–1.06			**1.16**	**1.06–1.26**
At least once a week			**2.01**	**1.65–2.45**			0.96	0.90–1.02			1.03	0.97–1.10			**1.21**	**1.11–1.32**
Daily			1.27	1.01–1.60			0.98	0.92–1.05			1.01	0.95–1.09			**1.25**	**1.14–1.37**
Newspaper use																
Not at all			1.00				1.00				1.00				1.00	
Less than once a week			**1.35**	**1.07–1.70**			**1.16**	**1.10–1.22**			0.99	0.93–1.05			**1.19**	**1.10–1.29**
At least once a week			**1.60**	**1.20–2.13**			**1.20**	**1.13–1.28**			0.99	0.93–1.05			**1.25**	**1.15–1.36**
Daily			1.10	0.73–1.67			**1.25**	**1.15–1.36**			0.95	0.88–1.04			**1.25**	**1.13–1.39**

Note: All models are additionally adjusted for age, gender, marital status, rural/urban location, literacy level, occupation, countries and survey year.

All the figures in bold have a p-value of <0.01.

HIV/AIDS Transmission Knowledge: HIV/AIDS transmission knowledge was also strongly associated with wealth and education among women, as shown in Model 1 of Table 3. However, these relationships were attenuated when media use was added to the model (Model 2). For example, after adjusting for mass media use, the associations for those in the most educated quintile (RR = 2.70; 95% CI: 2.45–2.98) with knowledge of HIV/AIDS transmission was attenuated (RR = 2.35; 95% CI: 2.13–2.60) (***Table 3***). In Model 2, HIV/AIDS transmission knowledge was associated with daily newspapers (RR = 1.31; 95% CI: 1.22–1.41), television (RR = 1.11; 95% CI: 1.06–1.17), and radio (RR = 1.07; 95% CI: 1.02–1.11) use. The analysis of men was not significant in either direction as the confidence interval crosses 1 in most cases except for less than once a week radio and daily radio.

HIV/AIDS Prevention Knowledge: As shown in Table 3, we found a similar trend of the wealth gradient and attenuation with the addition of media variables for prevention knowledge among women. HIV/AIDS prevention knowledge was associated with wealth and education. After adjusting for mass media use, the associations for those in the wealthiest quintile (RR = 1.17; 95% CI: 1.10–1.24) with knowledge of HIV/AIDS prevention was attenuated (RR = 1.06; 95% CI: 0.99–1.13). In Model 2, only the daily use of radio (RR = 1.20; 95% CI: 1.15–1.25) was strongly associated with increased knowledge of HIV/AIDS prevention. This association among men was more apparent than it was among women (***Table 4***).

Condom Use: The pattern of the influence of SES and the attenuation of its role with media exposure continued for condom use. We found wealth- and education-based gradients on condom use, and these gradients were attenuated once we accounted for mass media use (***Table 3***). Among women, the wealthiest group (RR = 2.00; 95% CI: 1.80–2.22) and those with 13 years or more of schooling (RR = 3.39; 95% CI: 2.89–3.97) were the most likely to use a condom during her last instance of sexual intercourse, but the relationships were attenuated among all classes when accounting for mass media use in the models. Those who use newspapers (RR = 1.28; 95% CI: 1.16–1.42), radio (RR = 1.23; 95% CI: 1.14–1.32), and television (RR = 1.22; 95% CI: 1.12–1.32) daily were more likely to use a condom at the last sexual intercourse. We found similar associations between media use and HIV prevention behavior, such as condom use, among men (***Table 4***).

**Figure 2:**
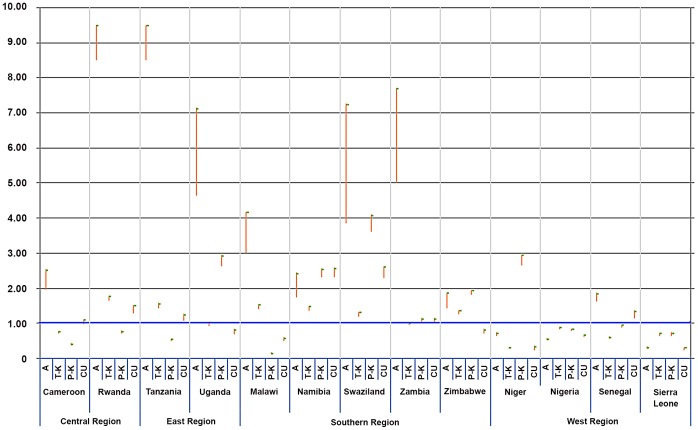
Relative risk and its 95% confidence interval for communication inequalities in media use for the relationships between socioeconomic inequalities and HIV/AIDS-related knowledge and condom use among thirteen sub-Saharan Africa countries and their regions. *Note 1*: A (HIV/AIDS awareness (A); T-K (HIV/AIDS transmission knowledge (T-K); P-K (HIV/AIDS prevention knowledge (P-K); CU (Last intercourse used condom (CU). *Note 2*: Each bar indicates relative risk and its 95% confidence interval for communication inequalities in media use for the relationships between socioeconomic inequalities and HIV/AIDS-related knowledge and condom use among thirteen sub-Saharan Africa countries and their regions. All figures are adjusted for potential confounders of this study and standardized by the average characteristics of the pooled population of all countries (Ref. = 1.00).

After standardizing the average characteristics of the pooled population of all countries as the reference (1.00), the associations between media use and HIV/AIDS-related knowledge and condom use which were shown in Table 3 and 4, were more significant in southern regions, such as Swaziland and Namibia, than in others (***Figure 2***). This probably means that profound inequalities in health communication still exist not only among individuals, but also within the country and region, despite mass media campaigns for HIV/AIDS control in those countries [27], [28].

## Discussion

Communication behaviors are often linked to an individual’s socioeconomic background and his or her knowledge and behavior relating to a specific issue. Our results indicated that the relationship between SES and HIV/AIDS-related knowledge and safe-sex behavior are moderated by the effect of media use. We found that social inequalities are associated with communication inequalities in sub-Saharan Africa. First, while there is substantial HIV/AIDS awareness among individuals at every socioeconomic level, increasing wealth and education are consistently associated with HIV/AIDS awareness, prevention and transmission knowledge, and condom use among both men and women. Education is particularly strongly associated with both forms of knowledge and behavior. This result is consistent with the results of other HIV/AIDS studies in sub-Saharan Africa, which have found a social gradient in the relationship between SES and HIV/AIDS [4], [5]. Second, these associations are attenuated when media use is added to the models. There may be profound inequalities in communication among different social groups in sub-Saharan Africa. Media use is positively associated with wealth and education in a gradient that is clear and convincing. Indeed, newspaper and radio use are significantly associated with SES (See ***Appendix S1 and S2 in File S1***). This observation is consistent with the notion that individuals of higher SES tend to gain more benefits from information resources than their counterparts of lower SES in LICs [28]. However, little is known about how media use may blunt the impact of SES in sub-Saharan African countries. Our observation is also consistent with the notion that individuals of any socioeconomic status can work to protect their own health if they can access the resources and information required to do so [14]. Lastly, the effects of mass media in this study vary by country and region. Southern countries, where HIV prevalence is high, also show stronger effects of media use compared to the other regions. This indicates that the effects of mass media campaigns and outreach programs in these high prevalence countries have been effective. Thus, we need to further reduce communication inequalities in order to improve the average level of HIV/AIDS knowledge and to promote condom use. Knowledge is a key variable influencing condom use according to the previous meta-analysis [29].

Several limitations of this study should be noted. The observational nature of the cross-sectional data prevents us from making causal inferences regarding the associations among socioeconomic variables, media use, and HIV/AIDS-related knowledge. However, the outcomes of interest in this study–the aspects of media use–are unlikely to precede the exposures under investigation, education and income, thereby reducing the possibility that the results would reflect reverse causation. In addition, previous studies demonstrating that media use leads to increased knowledge and healthy behavior support our assumptions regarding the ordering of the constructs [13], [30]. Concerning the generalizability of the study, more robust longitudinal studies are needed to establish a causal relation between media use and HIV/AIDS-related knowledge and behavior. Lastly, this study did not conduct media content analysis so it is not possible to determine which messages the study subjects received from their media use. Also, future research should explore the potential of mobile phones and how they can be harnessed for health promotion given their increasing penetration. Despite these limitations, the study is a novel use of thirteen nationally representative datasets created to study communication inequality as a driver of social inequalities in HIV/AIDS-related knowledge and behavior, with sensitivity to differences in country and region.

The findings of this study clearly point to an opportunity for mass media to promote HIV/AIDS knowledge and condom use to reduce the invidious impact of socioeconomic status on health. The caveat, however, is that inequalities in communication, specifically unequal use and exposure to different communication channels between higher and lower SES groups, has the potential to obstruct the promise of mass media. This calls for public health communication strategies that take into account these inequalities in order to overcome barriers across social classes. Thus, we need to apply media-based approaches to improve HIV/AIDS-related knowledge and condom use in sub-Saharan Africa while considering the differences in media use and exposure according to wealth and education. At the same time, we should pay attention to reducing communication inequalities among social groups and countries so as to achieve health equity on HIV/AIDS.

## Supporting Information

File S1 Appendix S1, Correlations among independent variables. P<0.01, a figure in bold caps. Appendix S2, Correlations among independent and dependent variables. P<0.01, a figure in bold caps.
(DOCX)
