# Supplementary material for: Effect of Media Use on HIV/AIDS-Related Knowledge and Condom Use in Sub-Saharan Africa: A Cross-Sectional Study
Source: PLoS One. 2013 Jul 12;8(7):e68359. doi: 10.1371/journal.pone.0068359 (PMC3709989; doi:10.1371/journal.pone.0068359)
Supplement: File S1 — Appendix S1, Correlations among independent variables. P<0.01, a figure in bold caps. Appendix S2, Correlations among independent and dependent variables. P<0.01, a figure in bold caps. (DOCX) [file pone.0068359.s001.docx]

**Appendix S1.** Correlations among independent variables

|  | Wealth | Education | Radio use | Television use | Newspaper use |
| --- | --- | --- | --- | --- | --- |
| Wealth | 1 | **0.451** | **0.363** | **0.550** | **0.373** |
| Educational attainment | | 1 | **0.313** | **0.466** | **0.561** |
| Radio use | | | 1 | **0.347** | **0.330** |
| Television use | | | | 1 | **0.429** |
| Newspaper use | | | | | 1 |

P < 0.01, a figure in bold caps

**Appendix S2.** Correlations among independent and dependent variables

|  | HIV/AIDS  Awareness | HIV/AIDS  transmission  knowledge | HIV/AIDS  prevention  knowledge | Last intercourse  used condom |
| --- | --- | --- | --- | --- |
| Wealth | **0.162** | **0.180** | **0.033** | **0.168** |
| Education | **0.205** | **0.244** | **0.061** | **0.277** |
| Radio use | **0.201** | **0.115** | **0.043** | **0.147** |
| Television use | **0.137** | **0.130** | **0.071** | **0.212** |
| Newspaper use | **0.138** | **0.196** | **0.053** | **0.276** |

P < 0.01, a figure in bold caps
